# Measuring the impact of pharmacoepidemiologic research using altmetrics: A case study of a CNODES drug‐safety article

**DOI:** 10.1002/pds.4401

**Published:** 2018-03-24

**Authors:** J.M. Gamble, Robyn L. Traynor, Anatoliy Gruzd, Philip Mai, Colin R. Dormuth, Ingrid S. Sketris

**Affiliations:** ^1^ Faculty of Science, School of Pharmacy University of Waterloo Waterloo Ontario Canada; ^2^ School of Pharmacy Memorial University of Newfoundland St John's Newfoundland & Labrador Canada; ^3^ Department of Community Health & Epidemiology Dalhousie University Halifax Nova Scotia Canada; ^4^ Ted Rogers School of Management Ryerson University Toronto Ontario Canada; ^5^ Faculty of Medicine, Department of Anesthesiology, Pharmacology, & Therapeutics University of British Columbia Vancouver British Columbia Canada; ^6^ Faculty of Health Professions, College of Pharmacy Dalhousie University Halifax Nova Scotia Canada

**Keywords:** alternative metrics, altmetrics, bibliometrics, citation metrics, knowledge translation, pharmacoepidemiology, research evaluation, research uptake, scholarly impact

## Abstract

**Purpose:**

To provide an overview of altmetrics, including their potential benefits and limitations, how they may be obtained, and their role in assessing pharmacoepidemiologic research impact.

**Methods:**

Our review was informed by compiling relevant literature identified through searching multiple health research databases (PubMed, Embase, and CIHNAHL) and grey literature sources (websites, blogs, and reports). We demonstrate how pharmacoepidemiologists, in particular, may use altmetrics to understand scholarly impact and knowledge translation by providing a case study of a drug‐safety study conducted by the Canadian Network of Observational Drug Effect Studies.

**Results:**

A common approach to measuring research impact is the use of citation‐based metrics, such as an article's citation count or a journal's impact factor. “Alternative” metrics, or altmetrics, are increasingly supported as a complementary measure of research uptake in the age of social media. Altmetrics are nontraditional indicators that capture a diverse set of traceable, online research‐related artifacts including peer‐reviewed publications and other research outputs (software, datasets, blogs, videos, posters, policy documents, presentations, social media posts, wiki entries, etc).

**Conclusion:**

Compared with traditional citation‐based metrics, altmetrics take a more holistic view of research impact, attempting to capture the activity and engagement of both scholarly and nonscholarly communities. Despite the limited theoretical underpinnings, possible commercial influence, potential for gaming and manipulation, and numerous data quality‐related issues, altmetrics are promising as a supplement to more traditional citation‐based metrics because they can ingest and process a larger set of data points related to the flow and reach of scholarly communication from an expanded pool of stakeholders. Unlike citation‐based metrics, altmetrics are not inherently rooted in the research publication process, which includes peer review; it is unclear to what extent they should be used for research evaluation.

**Key Points**

- “Alternative” metrics, or altmetrics, can complement traditional citation–based measures to assess the reach, uptake, and short‐term impact of drug‐safety articles.
- Altmetrics may allow for a broader view of research uptake, as they process data related to the flow and reach of activity and engagement from both scholarly and nonscholarly communities.
- There are a variety of tools available to acquire altmetrics for assessing research impact; potential users should understand each tool's unique benefits and limitations.
- Pharmacoepidemiologists may use altmetrics to evaluate and better understand the extent of online attention of their scholarly work, as well as to identify potential audiences and collaborations in drug‐safety and ‐effectiveness research.
- Further work is needed to explore data quality issues and determine the accuracy and interpretations of altmetrics within the context of drug‐safety and ‐effectiveness research to capture meaningful impact.

## 1 INTRODUCTION

Findings from pharmacoepidemiology studies are often relevant to a broad audience including scientists, healthcare professionals, policy makers, industry, and the public. Research funders, such as the National Institutes of Health, the European Research Council, and the Canadian Institutes of Health Research (CIHR), are also keen to understand the impact of the research they fund.^1^ Although citation‐based author level (eg, *h*‐index^2^); article level (eg, cumulative number of citations per article); and journal level (eg, journal impact factor) bibliometrics have served as the mainstay of measuring scholarly impact for decades, altmetrics are increasingly becoming recognized as a complementary measure of research impact in the age of the social web.^3^, ^4^, ^5^, ^6^, ^7^, ^8^

Alternative metrics, or altmetrics for short, trace the flow of scholarly communication across a diverse range of research outputs, among a broad audience and in essentially real time.^9^, ^10^, ^11^ Importantly, altmetrics can capture previously hidden elements of engagement with research outputs from both scientific and nonacademic audiences.^10^, ^12^ Today, a researcher may download the PDF of an article, save it to her online reference manager, discuss the article on social media and blogs, and provide comments or formally recommend the article online post‐publication in an academic social network (eg, F1000, Mendeley, ResearchGate, Academia.edu). The historical equivalent may have been to read an article in a print journal, store a copy in an office file, engage in informal hallway conversations, and perhaps comment on or endorse the article in a conference presentation. The use of altmetrics continues to grow and is becoming more prominent in some fields (eg, information, medical, and biomedical sciences)^13^, ^14^, ^15^, ^16^, ^17^, ^18^ but has been used less frequently by pharmacoepidemiologists to date.^19^, ^20^ There is limited information on how individual articles on population‐level drug‐safety and ‐effectiveness research diffuse through the web and whether the data derived can be useful in determining patterns of knowledge translation of drug‐safety issues. Altmetrics may be a promising approach for better understanding, planning, and implementing efforts to translate knowledge from observational drug‐effect studies to policy makers, healthcare professionals, industry, and the public.

This article will provide an overview of altmetrics, including where they may be obtained from, their benefits and limitations, and their role in assessing pharmacoepidemiologic research impact. We informed the following overview by compiling relevant literature from the field of altmetrics in health research, with a particular focus on its use in pharmacoepidemiology. We worked with a librarian at Dalhousie University to develop and implement search strategies in health research databases (PubMed, Embase, and CINAHL), without restrictions on publication year and using the following terms: altmetric* OR infodemiology OR (metric* AND social media). We searched the grey literature (websites, blogs, and reports) and hand‐searched journals featuring altmetrics (e.g., PLoS One altmetrics collection) and the reference lists of key sources, including those already known to us or identified in our database search. We present a case study of the altmetrics for a study conducted by the Canadian Network for Observational Drug Effect Studies (CNODES) on higher potency statins and the risk of incident diabetes^21^ to further illustrate how altmetrics tools and techniques can be applied in drug safety (Text Box 1).

Box 1 **A case study for using altmetrics in pharmacoepidemiology**

CNODES is a nationally distributed network of researchers and data centers using collaborative, population‐based approaches to study drug‐safety and ‐effectiveness. CNODES is funded by the CIHR and is a collaborating center of the Drug Safety and Effectiveness Network.^39^ CNODES' knowledge translation efforts follow a rigorous dissemination strategy to target actionable messages from its studies to various stakeholders. Altmetrics are one way of indicating impact, specifically by measuring the extent to which CNODES' research has reached and been taken up by these target audiences. To examine this uptake, we collected altmetrics data for a CNODES study^21^ on October 1, 2014, 4 months following its publication, using 4 complementary approaches.

**Figure 1:**
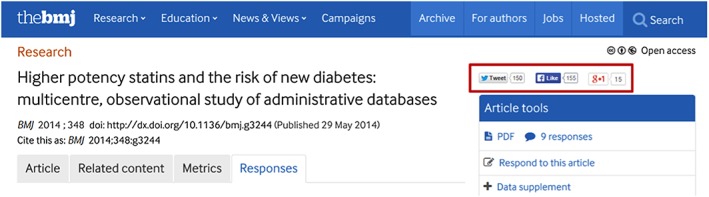
Social media mentions and “likes” for Dormuth et al^21^ on BMJ website. (http://www.bmj.com/content/348/bmj.g3244) [Colour figure can be viewed at http://wileyonlinelibrary.com]

**Figure 2:**
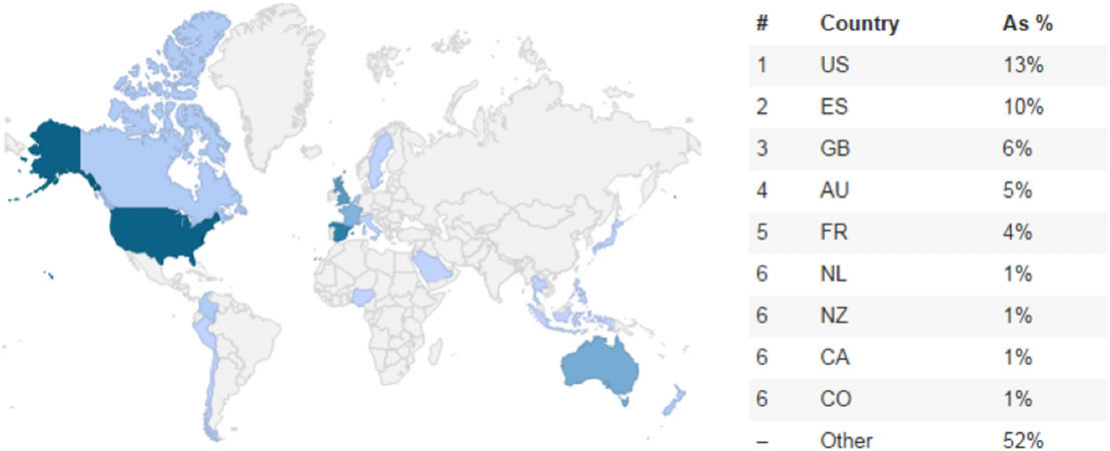
Geographic breakdown of Twitter user activity related to Dormuth et al.^21^ (https%3A%2F%2Fbmj.altmetric.com%2Fdetails%2F2392997%0A) [Colour figure can be viewed at http://wileyonlinelibrary.com]

**Figure 3:**
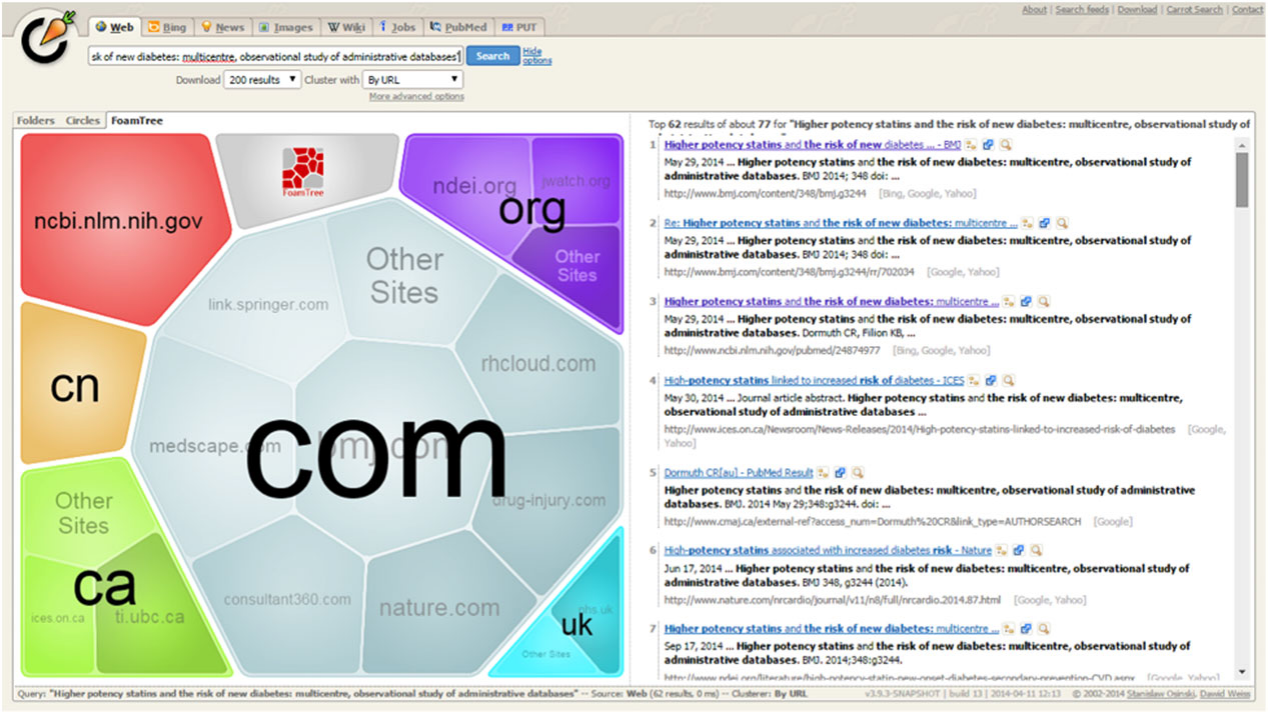
Websites retrieved by Carrot2.org that mentioned the title of Dormuth et al^21^ in full. Pages are grouped by their top‐level domain names. The size of each cluster indicates the number of websites within that cluster, relative to other clusters [Colour figure can be viewed at http://wileyonlinelibrary.com]

**Figure 4:**
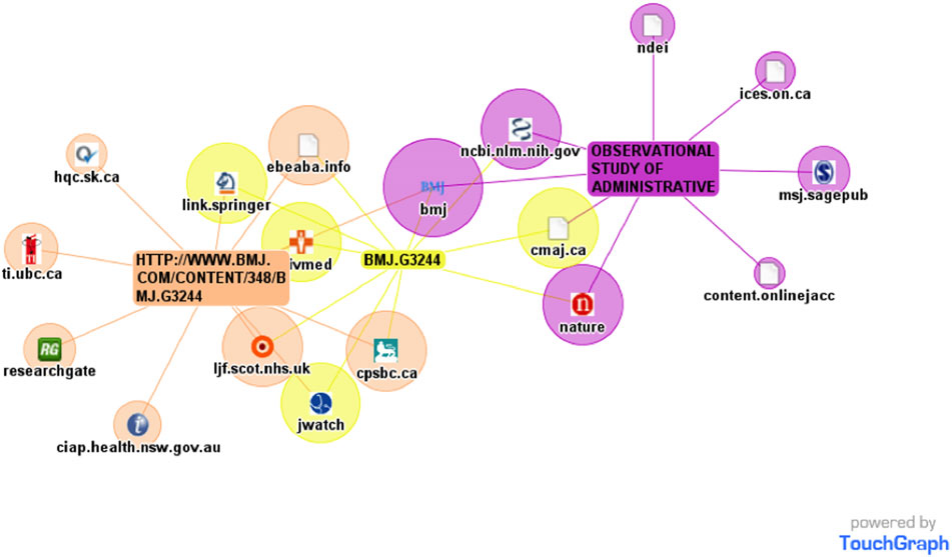
Network of websites retrieved by Touchgraph.com that mentioned or linked to Dormuth et al^21^ [Colour figure can be viewed at http://wileyonlinelibrary.com]

Source 1 *BMJ Article Publication Page:* Dormuth et al^21^ received 9 comments, with the majority (6 of 9) posted within a month of online publication. The link to Dormuth et al^21^ was shared on Twitter 150 times, “liked” on Facebook 155 times, and shared 15 times on Google+ (Figure 1), indicating the extent to which its readers or potential readers found this article interesting and/or relevant to their work. Appendix S1 shows the number of times Dormuth et al^21^ was accessed and downloaded from the BMJ website during first 4 months following the publication date.
Source 2 *Altmetric.com:* Altmetric.com found that Dormuth et al^21^ was mentioned by 116 Twitter users, 7 Facebook users, 2 Google+ users, and 2 Weibo (Chinese social networking site) users. It was also bookmarked by 19 Mendeley and 2 CiteULike users (both scholarly bookmarking services). On the basis of these stats, Dormuth et al (2014) scored higher than 98% of other articles published around the same time §
Altmetric.com tracked 76 490 articles from any journal 6 weeks published before or after May 29, 2014. and scored higher than 95% of BMJ articles of the same age. The numbers reported by Altmetric.com differ from those reported on the BMJ publication page in part because the BMJ numbers demonstrate how many people shared the article by directly clicking on the share icon on their page; they do not account for instances when people mentioned the article using other methods (eg, by posting a Twitter message directly from their account). Altmetric.com only tracks instances when the article is mentioned by a unique persistent identifier, such as its DOI number, and will therefore miss any instances when the article is mentioned without referencing an identifier which is tracked. Altmetric.com also provides additional information about the Twitter readership base by classifying users into broad categories and noting their geographic location. Twitter readership of Dormuth et al^21^ included members of the public (78%), healthcare professionals (16%), researchers (3%), and science communicators (1%). While these figures need to be accepted with caution, given the computational challenges of classifying Twitter users by roles and the fact that users may belong to multiple categories, they generally suggest that the article topic is of interest to a broader range of individuals than merely other researchers. Dormuth et al^21^ attracted international attention whereby the largest number of Twitter users are from the US, followed by Spain, the UK, and Australia (Figure 2) (It may be important to note that map data are skewed towards countries where Twitter is popular and available.).
Source 3 *Mainstream media coverage:* We reviewed altmetrics data from the mainstream media, specifically CTV News network coverage, on May 29, 2014 (http://bit.ly/2gzBiqU). This story was shared 312 times overall using the “share” icon and was recommended 711 times on Facebook. To further assess the potential reach of the original article through this CTV story, we examined how many Twitter users who shared it are considered to be “influential” themselves; in other words, those who have a large follower base and whose messages are often reposted. According to the website Topsy.com ¶
One of the tools we used to assess the context of Twitter accounts, Topsy.com, was discontinued in December 2015, reinforcing the ever‐changing landscape of altmetrics tools. that tracks social media mentions, among those Twitter users who shared the link to the CTV story, 7 members (primarily those with a CTV‐related account and/or who are health‐related writers) are considered “influential”.
Source 4 *Web search results:* Carrot2.org retrieved websites that mentioned the full article title and automatically grouped up to 200 of the most relevant results based on their top‐level domain name. The top 3 domains were .com (n = 35), .org (n = 8), and .ca (n = 8) (Figure 3). Touchgraph.com identified a network of websites that mentioned or linked to Dormuth et al,^21^ as well as additional resources related to these websites (Figure 4).

There are several limitations with our case study. First, we discussed the altmetrics of the article but did not specifically examine the nature and effect of the public relations activities of the BMJ, the Lady Davis Institute of McGill University (site of CNODES' central office), nor the universities at other CNODES sites. Second, we did not examine the context in which the social media discussions occurred or qualitative information about the social media content (eg, accuracy or positive and negative emotion to words). While we know that the article was accessed, we do not know if or how the information was used.^40^ For example, we could not determine if patients contacted a healthcare professional as a result of media coverage or other online content. We also were not able to determine why individuals linked to our article, including whether it was related to their interest in the BMJ, statins, adverse drug reactions, or other issues. We determined the number of tweets but not the names of those who were tweeting or the number of retweets and new followers. Third, our time was limited to 4 months since the article was released, and we did not assess temporal trends such as seasonality within the period of data capture. Lastly, we did not examine the credibility, quality, or funding source of the blogs and examined only English language sources.

### 1.1. Defining altmetrics

The term altmetrics was coined on Twitter in 2010 by Jason Priem,^22^ who subsequently defined them as “the study and use of scholarly impact measures based on activity in online tools and environments.”^10^ More recently, the National Information Standards Organization (NISO) has defined altmetrics as a “broad term that encapsulates the collection of multiple digital indicators related to scholarly work … [that] are derived from activity and engagement among diverse stakeholders and scholarly outputs in the research ecosystem, including the public sphere.”^23^ Altmetrics are a subset of webometrics or cybermetrics in that they focus on measuring the online engagement with research products through social media, reference managers, blogs, etc.^10^ Altmetrics are concerned with online‐ or web‐based sources of measuring scholarly activities and use the technological infrastructure of the modern web (ie, Web 2.0, defined as websites that underscore user‐generated content, interaction, and collective knowledge production and exchange^24^, ^25^, ^26^) to capture information on the social immediacy and visibility of many types of research outputs in nearly real time. They are not specific to a level of aggregation; they may be applied at the article, author, journal, funder, geographical region, subject area, or institutional level to contribute to the assessment of research impact.^27^

Scientometricians and research evaluators have been using nontraditional sources of data to track scholarly impact such as acknowledgements, patents, mentorships, news articles, and use in syllabi.^10^ The novelty in altmetrics data sources is not that they create new scholarly practices but that they enable formal and informal communication to occur in a traceable format over the web. Whereas citation‐based metrics are centered on the peer‐reviewed manuscript, altmetrics include metrics on many types of research outputs or artifacts that are traceable on the web *
For a comprehensive list of research outputs/artifacts, visit https://sites.google.com/a/niso.org/scholarlyoutputs/. including peer‐reviewed manuscripts; datasets; software code; blog posts; videos; presentations; and shares, likes, and posts made on social media.^9^ Indeed, it is the creation of social networks on Web 2.0 that allows for communication among all knowledge users including scientists, healthcare professionals, policy‐makers, and the public.^24^ Citation‐based metrics fail to capture this breadth of an audience, which is important, given it is estimated that only about 15% to 20% of United States–based scientists have published a peer‐reviewed article^28^ and a small core group of scientists is responsible for publishing a large proportion of articles.^29^

### 1.2. Altmetrics tools and data sources

There are a variety of tools—known as altmetrics data aggregators—available for researchers, institutions, and funding agencies to acquire altmetrics for assessing the impact of their research.^30^ Examples of altmetrics data aggregators are Altmetric.com (altmetric.com), Impactstory (impactstory.org), Plum Analytics (plumanalytics.com), KUDOS (growkudos.com), and Researchfish (researchfish.net). Altmetrics data aggregators capture online digital behavior from a diverse set of data sources (Table 1) by tracking a unique online persistent identifier for the research output or increasingly using text‐mining algorithms. Persistent identifiers for research outputs include digital object identifier (DOI), PubMed ID, arXiv ID, SSRN ID, ClinicalTrials.gov, and ORCID. †
For a complete list of unique, persistent identifiers for research outputs, visit https://sites.google.com/site/nisopersistentids/. The types of altmetrics generated are primarily quantitative data consisting of counts of HTML views, PDF downloads, social media mentions, Wikipedia and blog citations, journal comments, etc. Specific social media platforms used for altmetrics include social networking (e.g., Facebook and ResearchGate); social bookmarking and reference management (e.g., Mendeley, CiteULike, Zotero, and RefWorks); social data (e.g., datasets, software code, presentations, figures, and videos); sharing (e.g., Figshare, GitHub and YouTube); blogging (e.g., ResearchBlogging and WordPress); microblogging (e.g., Twitter and Sina Weibo); wikis (e.g., Wikipedia); and social recommending, rating, and reviewing (e.g., Reddit and F1000Prime). News media, policy documents, library holdings, and download statistics may also be considered relevant altmetrics sources.^31^ In addition to measuring “the quantity of attention received,” some altmetrics data aggregators integrate the “quality of attention” (e.g., a news story counts for more than a Facebook post, and attention from a researcher counts for more than attention from an automated Twitter bot).^32^ However, because of the proprietary nature of many altmetrics tools, the exact nature of the scoring algorithms is not always disclosed.

**Table 1:** Examples of potential altmetrics data sources

Types of Data Source	Data Sources
Social networking	Facebook, ResearchGate, Google+, LinkedIn
Social bookmarking and reference management	CiteULike, Mendeley, Zotero
Social data sharing	Figshare, GitHub
Blogging	ResearchBlogging, WordPress
Microblogging	Twitter, Sina Weibo
Wikis	Wikipedia
Social recommending, rating, and reviewing	F1000Prime, Reddit, Publons, Pubpeer, journal comments
Other	News media, policy documents, library holdings, download statistics

Where would a researcher start if she was interested in obtaining altmetrics for her most recent article or perhaps all her articles? One place to start is the journal publisher website. Many publishers, including Biomed Central, BMJ Journals, Dove Press, Frontiers, The Lancet Journals, Nature, PLoS Journals, Taylor and Francis, and Wiley, have integrated in‐house or proprietary altmetrics tools (such as Altmetric.com or Plum Analytics ‡
Altmetric.com is a product of Digital Science^33^; Plum Analytics was acquired by Elsevier^34^ in February 2017.) for all or some of their journals. Moreover, many journals as well as news media outlets and blogs offer their visitors several ways to share and discuss individual articles. The BMJ, where Dormuth et al^21^ is published (see Text Box 1), offers a metrics tab, the ability to share the link to the article on social media platforms including Twitter, Facebook, and Google+, and a responses tab (Figure 1) the latter is where electronic letters to the editor are posted. Comments are not anonymous, which helps to attract well‐articulated and detailed responses from peers (on average of about 270 words), many of which also include relevant references. Visitors may “like” a comment that they read on the BMJ responses tab, adding interactivity to the website and allowing readers to express their support for a comment.

Many of the altmetrics tools (Impactstory and KUDOS) have web browser–based applications whereby a researcher enters a persistent author level (ORCID) or article level (DOI) identifier and a set of digital indicators will be provided. Altmetric.com has several products for researchers including a “bookmarklet” that directly integrates into a web‐browser bookmark bar and “badges” that may be used for personal webpages or curriculum vitae. They also provide access to an application programming interface (API), which enables researchers to request specific content from the Altmetric.com servers, allowing the data to be analyzed and presented directly by the researcher. For example, researchers may use Altmetric.com's API to obtain data for a research study. Although some of these tools are free, access to the full suite of many of the products and tools used by institutions and publishers requires subscription.

Researchers may also explore the uptake and spread of their work by using clustering (e.g., Carrot2.org) and visual (e.g., Touchgraph.com) search engines to identify additional web resources that mention the full article title and view the interconnections between resources. Carrot2.org is an open‐source clustering search tool that automatically organizes search results from Google, Yahoo, Ask, and Bing into thematic categories based on a few broad (user‐selectable) criteria such as page content or common domain name. Touchgraph.com is a visual search engine that represents search results in the form of a network of hyperlinks between retrieved web resources. These connections offer a glimpse into the interconnections between seemingly disparate websites. The network shows which popular websites link to the target article directly, which only mention the title of the article, and which do both. Of particular interest are the websites that do not include a link back to the article. The chance that the website's visitors will find and access the article full text on their own is reduced if a direct link to the cited article is not provided. Another use of the TouchGraph network visualization is to find other websites that might be interested in your research because they are related to or are linked with sites that have already engaged with your work.

## 2 THE UTILITY OF ALTMETRICS FOR PHARMACOEPIDEMIOLOGISTS

The NISO Alternative Assessment Metrics Project^23^ suggests 3 overarching themes for users of altmetrics: showcasing achievements, research evaluation, and discovery. For example, pharmacoepidemiologists may use altmetrics to highlight the reach, engagement, and influence of their work on their website, curriculum vitae, or tenure and promotion packages. Given that high levels of online activity may be an early indicator of a highly cited article, altmetrics are particularly useful for recently published material.^6^, ^11^ Eysenbach^35^ noted that when comparing altmetrics with citation data from Scopus and Google Scholar for a subset of the Journal of Medical Internet Research, highly tweeted articles were 11 times more likely to be highly cited than less tweeted articles.^35^ However, the correlation between Twitter activity and citations is highly variable, and the totality of the literature does not support a significant correlation.^36^ At the same time, the number of blog posts mentioning a publication has been shown to increase the likelihood of a paper receiving a new citation by nearly 37% in the field of Health Professions and Nursing.^37^

Importantly, altmetrics allows tracking of research uptake beyond the peer‐reviewed manuscript including op‐eds, blogs, editorials, post‐publication peer review (e.g., f1000.com); software (e.g., GitHub.com); knowledge translation products such as drug information tools; and other online content related to research (e.g., videos, posters, and slide decks from presentations). For example, pharmacoepidemiologists could post the programming code used for their analysis on GitHub and then measure the interest in the code by using altmetrics. One could also use altmetrics to provide evidence of the research impact of completed projects and strengthen subsequent applications for grant support. Similarly, altmetrics may be used in writing reports for funding agencies, departments, or other institutional bodies who are often interested in the broader societal impact of research. Journal‐level altmetrics provide evidence of audience exposure that is relevant information when deciding where to submit articles for publication. Likewise, the discovery of potential collaborators and influential research within pharmacoepidemiology are facilitated using altmetrics. Furthermore, altmetrics may be used to evaluate the public's understanding, knowledge, attitude, and beliefs about drug effects.^38^ For example, pharmacoepidemiologists, drug regulators, and agencies such as the US Food and Drug Administration may use social media analytics to understand how the public is engaging with the latest information related to risk/benefit balance of new drugs and risk mitigation plans and to expand bidirectional communication opportunities between these groups and their stakeholders. Altmetric tools may also be used to understand the interest and concerns of multiple stakeholders.^38^ To further demonstrate the utility of altmetrics for measuring the immediate scholarly impact of a pharmacoepidemiologic article, we examined the altmetrics of a study published by CNODES^39^ that evaluated the association between high‐potency statin use and the risk of developing diabetes^21^ (Text Box 1 ).

### 2.1 Considerations when interpreting altmetrics

Altmetrics offer solutions to track previously hidden avenues of scientific communication across scholarly and broader communities. A significant benefit of altmetrics is the speed or responsiveness for capturing knowledge user engagement with a diverse set of research outputs. Altmetrics often pick up activity within days compared to months or years with traditional citation metrics.^41^ Despite these potential benefits, there are several methodological issues surrounding the use of altmetrics.

First, a significant limitation of altmetrics is the inability of any single metric to disentangle quality, importance, relevance, and intent of the research output. Although distinctions among these elements are not always clear with traditional citation–based bibliometrics, citation‐based metrics are an integral part of the research process and are accepted as an important measure of scholarly impact—they are directly relevant and rooted in scholarly activities, including peer review. For example, pharmacoepidemiologists publish their work in reputable academic journals and directly cite supporting literature or in some cases cite the limitations of the previous literature. Indeed, publishing and citing literature within publications are the core activities in which a scientist rigorously engages with the community. In contrast, altmetrics measure activities that are not intrinsically embedded in the research process. Indeed, altmetrics appear to be measuring something complementary to citation‐based metrics. The correlation between altmetrics and citation‐based metrics varies by data source and is higher for sources used primarily by academics. Bornmann^36^ meta‐analyzed studies measuring correlation coefficients between 3 common sources of altmetrics: microblogging, blogging, and reference managers. Pooled correlations between altmetrics and citation‐based metrics across multiple studies were low for the microblogging platform Twitter (pooled *r* = 0.003) and low for blogs (pooled *r* = 0.12), with bookmarking in online reference managers having the highest correlation (pooled *r* = 0.37). Notably, there was a substantial amount of heterogeneity among the correlation studies (*I* ^2^ > 99% for all 3 meta‐analyses). Given the broader audience captured in altmetrics, higher levels of activity may simply reflect public interest, “buzz,” or popularity.^42^ The rise of social bots may further exacerbate the problem of undue attention to scholarly work that otherwise may not garnish such attention. Moreover, Robinson‐Garcia et al^43^ recently found that many tweets in dentistry journals were devoid of original thought and reflected mechanical bot‐like behavior, indicating that altmetrics based on tweets should be interpreted with caution if used for research evaluation.

Second, commercialization interests may be at play for altmetrics, as with traditional bibliometrics. For example, increasing the volume of posts, mentions, and likes on social media sites of positive experiences is of inherent commercial interest to the site. Similarly, companies that provide altmetrics have a commercial incentive to promote their value to librarians, institutions, and research funding bodies. Social media activity may be exploited by parties with a potential competition of interest such as pharmaceutical companies, advocacy groups, or individual researchers.^43^ Moreover, the rapid rise of predatory scientific publishing entities adds further noise to online activity.^44^ These predatory journals may be mistaken for legitimate journals by scholars and the public.^45^ Patients engaging in social media may be targets for promotion of health products or disease‐based advertising. Pharmaceutical regulators may also be interested in analyzing altmetrics for purposes of tracking online activity of their own outputs, such as advisories about new safety signals or drug product monograph updates. When interpreting altmetrics, identifying the source of online activity and classifying whether there are potential conflicts is particularly relevant in pharmacoepidemiology. It will be important for altmetrics data sources aggregators to work with relevant academic groups, editors, and others to develop methods to identify trusted and evidence‐based sources of knowledge, as well as sources with a real or perceived conflict of interest.

Third, gaming and manipulation are theoretically possible by the creation of false data through fake accounts and automation of downloads, tweets, posts, likes, etc.^6^, ^46^, ^47^ Although the notion of false positive hits on social media sites has partially been solved by the advertising industry whereby algorithms can identify patterns suggesting manipulation, there are many potential gaming scenarios that are not easily detectable.^48^ For example, antispam and antigaming algorithms are used by Google, Wikipedia, and Twitter to identify spurious and nefarious activity; however, not all suspicious activity can be automatically detected, and manual curating is still used.^48^ As mentioned, social media bots may inflate the online attention of online scholarly work. Indeed, Robinson‐Garcia et al^43^ found that bots accounted for 2.4% of tweets by US‐based accounts in dental journals. Another area where manipulation may occur is when readers vote to “like” online content. This feature is very ambiguous. What does a “like” mean for a comment that consists of several hundred words? Does the person support the comment in principle or do they “like” an argument presented? Since anyone on the web can “like” something without registering on the website, we believe that this feature is prone to gaming. Altmetrics.com is working towards greater transparency in both this issue, as well as its scoring algorithm.^49^

Fourth, there are many data quality issues surrounding altmetrics that may result in systematic error. Accuracy, consistency, and replicability of altmetrics data are cited as main issues.^50^ Data quality is also dependent on understanding the type of user engaging in research through social media. Certain altmetric tools differentiate between scholarly and public engagement through stratification of data sources. For example, scholars may tend to download PDFs, whereas the public may view HTML pages. More research is required to test the validity of these types of approaches. Ambiguity and redundancy may also occur when multiple versions of the same research output exist; altmetric data aggregators will typically not be able to distinguish between a preprint and postprint version of an article. Similarly, author disambiguation may be difficult given the lack of standard unique identifiers for specific researchers. ORCID (orcid.org) is one solution to this problem, although uptake has been slow to date.

Online behavioral patterns differ across disciplines in respect to the level of online engagement that will create disparities in the volume of altmetrics data generated. Social media behavior has also changed over time and with more recently published articles. Behavioral patterns may also differ across languages. Therefore, to allow for cross‐field and ‐time comparisons, altmetrics data must be normalized.^51^, ^52^, ^53^ Tested approaches have included a process for normalizing Twitter counts at the journal level,^52^ field‐normalized indicators based on Mendeley data,^51^ and normalization of Mendeley reader counts based on an established citation‐count normalization method.^53^ How to best distinguish different meanings between content‐rich (e.g., blog posts and Wikipedia) and content‐poor (e.g., Facebook shares or likes) data is also unclear. Consistency of view is another concern among the altmetrics community.^54^ Both raw counts and aggregate scores are presently used, with substantive variation in process and composition of aggregate scoring.

Our case study (Text Box 1) may have had specific features that made it highly accessed. The BMJ is one of the most highly cited journals in medicine (impact factor of 16.4 in 2013), and many blogs may focus on highly cited journals.^5^ Ioannidis^55^ suggests the most influential articles are concentrated in a minority of scientific journals, such as the BMJ. The article was also published open access; uptake, as measured by altmetrics, may be higher for open access publications.^56^, ^57^ In addition, the topic of our article was an adverse effect of statins, which are prescribed to millions of individuals worldwide.^58^

The development of data measurement standards would help improve data quality issues. Currently, there are no formalized standards such as a standard classification scheme for altmetrics data sources. In general, altmetric data aggregators group data sources into categories such as viewed, saved, discussed, and recommended. However, variations exist in the data sources within each category and how data sources are grouped into scholarly and nonscholarly sources. Lin and Fenner^59^ have reviewed the article‐level classifications of the leading altmetrics data aggregators. NISO has started to publish a series of outputs from its alternative assessment metric project that outlined best practices for the role of altmetrics across various uses.^23^, ^60^

Lastly, it is important to note that existing tools for generating altmetrics continue to evolve in the depth and breadth of their data sources; their classification schemes and the tools available to researchers, institutions, and publishers. For example, there may be limited historical data, which may vary between public (ie, free) and licensed APIs; the latter is often required for full access to real‐time and historical data. The number of data sources and text‐mining algorithms used to search for persistent identifiers changes over time, which limits the ability to analyze time trends and to compare altmetrics across different data aggregation tools. Moreover, privacy settings for social media and pay walls for journal publishers and media outlets may change over time, leading to variation in capturing certain types of altmetrics data.

Our case study provides several lessons for the role of altmetrics in pharmacoepidemiology. We provide an example of using altmetrics to measure the short‐term research impact of a drug‐safety study. Dormuth et al^21^ was mentioned hundreds of times by a wide range of online users (e.g., individual users and professional organizations), suggesting a high level of early online interest among members of the public and the medical community, in particular. Indeed, 4 months following the publication of Dormuth et al,^21^ it had generated a disproportional amount of online buzz in comparison to its peers. The uptake of the article began almost immediately following publication. Our timeline is similar to other articles that are most tweeted on the first day and most blogged about in the first month.^6^ Our article was in the top 5% of all articles ranked by the amount of attention compared to other articles in the BMJ.

Our case study also illustrates the way in which altmetrics can be used for formative and developmental evaluation and to determine which organizations could be “receptor site” targets (ie, to reach specific stakeholder audiences) for future articles to quickly communicate with other researchers, health professionals, decision makers, and the public. Academics can learn to use altmetrics to complement other knowledge translation strategies, both with the public and with other researchers.^5^, ^61^, ^62^ For example, we identified several organizations and individuals who are interested in this specific work of CNODES. They represent important members of CNODES' broader receptor community including, but not limited to, the mainstream media, health writers and bloggers, information resources for health professionals, and patient‐focused organizations such as the National Diabetes Education Initiative.

## 3 CONCLUSIONS

Altmetrics is increasingly being used to measure the scholarly impact of research within and beyond the scientific community. Although there are many potential benefits for using altmetrics, we have pointed out several concerns which require clarification. Indeed, as altmetrics become more popular and accepted, they may no longer be considered ‘alternative'. Our case study demonstrates that altmetrics, even in its current state, can complement traditional citation‐based measures to assess the short‐term impact of a drug‐safety article. As Bornmann (2015) concludes, future studies need to also focus on the potential of altmetrics to measure broader impacts of research, beyond academia.^36^ The rapid uptake and broad reach of information demonstrate its potential to provide drug benefit/risk information to many stakeholders. Further work is needed to explore data quality issues and determine the accuracy and interpretations of altmetrics within the context of drug safety and effectiveness research. Altmetrics could also be employed to document collaborations within pharmacoepidemiology research teams, such as CNODES and its alumni, as well as to determine future collaborations. Our altmetrics analysis identified which organizations and individuals are interested in this drug safety article. In future, this audience could be specifically targeted to more effectively and efficiently disseminate knowledge from future drug safety studies. Finally, we encourage pharmacoepidemiologists who are interested in utilizing altmetrics to evaluate the impact of their research to work with individuals with expertise in the information sciences and social media studies.

## Supporting information


**Appendix S1**. Number of times Dormuth et al. (2014) was accessed and downloaded from BMJ website since the publication date on May 29, 2014 to October 1, 2014.
