# Supplementary material for: Measuring the impact of pharmacoepidemiologic research using altmetrics: A case study of a CNODES drug‐safety article
Source: Pharmacoepidemiol Drug Saf. 2018 Mar 24;29(Suppl 1):93–102. doi: 10.1002/pds.4401 (PMC7004200; doi:10.1002/pds.4401)
Supplement: Supplementary file 1 — Appendix S1. Number of times Dormuth et al. (2014) was accessed and downloaded from BMJ website since the publication date on May 29, 2014 to October 1, 2014. [file PDS-29-93-s001.docx]

**Supplementary Material**

**Appendix S1.** Number of times Dormuth et al. (2014) was accessed and downloaded from BMJ website since the publication date on May 29, 2014 to October 1, 2014.
